# Human papillomavirus (HPV) prevalence and associated risk factors in women from Curaçao

**DOI:** 10.1371/journal.pone.0199624

**Published:** 2018-07-13

**Authors:** Desiree J. Hooi, Birgit I. Lissenberg-Witte, Gemma Kenter, Maurits N. C. de Koning, Igor Gomes Bravio, Kim Ardts, Suhaina Kleinmoedig, Edlyn Benita, Herbert M. Pinedo, Johannes Berkhof, Wim G. V. Quint, Chris J. L. M. Meijer

**Affiliations:** 1 Department of Pathology, VU University Medical Centre, Amsterdam, the Netherlands; 2 Department of Epidemiology and Biostatistics, VU University Medical Centre, Amsterdam, the Netherlands; 3 Department of Gynaecology and Oncology, VU University Medical Centre, Amsterdam, the Netherlands; 4 DDL Diagnostic Laboratory, Rijswijk, the Netherlands; 5 Fundashon Prevenshon, Willemstad, Curaçao; 6 Department of Pathology, Analytic Diagnostic Centre (ADC), Willemstad, Curaçao; Rudjer Boskovic Institute, CROATIA

## Abstract

**Background:**

In the Caribbean region, a notable difference in HPV-prevalence and genotypes distribution between the islands is observed. Recently we found in Curaçao a low incidence of HPV16 and 18 in cervical cancer compared to the standard world population. We aimed to determine HPV-prevalence, HPV-genotype distribution and associated risk-factors in women from Curaçao.

**Methods:**

5000 women aged 25–65 years were randomly selected from the national Population Register. HPV was detected by means of GP5+/6+PCR EIA and GP 5+/6+amplimers from HPV-positive samples were genotyped with a reverse hybridisation assay. We also collected personal data and data on risk-factors.

**Results:**

1075 women were enrolled in the study. Overall HPV-prevalence was 19.7%. Most frequent genotypes were HPV16 (2.3%), 35 (2.1%) and 52 (1.8%). Twenty-seven women detected with abnormal cytology (i.e.≥ASC-US) were referred for biopsy. In women with normal cytology (n = 1048), HPV-prevalence was 17.9% and the most common high-risk HPV (hrHPV)-types were HPV35 (2.0%), 18 (1.8%), 16 (1.5%) and 52 (1.5%). The highest HPV-prevalence (32.8%) was found in the age-group: 25–34 (n = 247). HPV positive women started sex at a younger age (p = 0.032).

**Conclusions:**

HPV-prevalence in the overall population is high and HPV16 was the most common genotype followed by 35 and 18. In women with normal cytology HPV35 is the most common genotype followed by HPV18, 52 and 16. The high HPV-prevalence (32.8%) in women of 25–34 years argue for introduction of cervical cancer prevention strategies. HPV-type distribution found in Curaçao should be taken into account when considering the choice for prophylactic vaccination.

## 1. Introduction

In the Caribbean region the cervical cancer incidence is high, mainly because of the lack of a structured prevention strategy.[1, 2] Although no formal age standardised Rate (ASR) of cervical cancer has been published, with a registered population of nearly 157.000 in 2015 (National department of Statistics in Curaçao), the incidence is estimated to be 13.4 per100, 000 women per year over the period 2008–2014 (C.M.D. Coronel, Pathologist, personal communication).

The Caribbean population is known for its unique diverse ethnic distribution consisting of Afro descendants mixed with other ethnicities settled in the region.[3] Published Caribbean data about HPV in the general population are limited and interpretation of the data is hampered by the small size and lack of age stratification of the populations investigated. These publications describe a high overall HPV prevalence, with non- HPV16 and 18 genotypes as the most common types.[1,4]

Three HPV prophylactic vaccines are currently registered: a bivalent vaccine against HPV16 and 18, a quadrivalent vaccine against HPV16 and 18 with an additional coverage of low risk HPV (lrHPV) 6 and 11, and a nonavalent vaccine which, in addition to the 4 types in the quadrivalent vaccine, covers high risk HPV (hrHPV) types 31, 33, 45,52 and 58.[5]

When considering cervical cancer prevention strategies i.e. population based screening and prophylactic vaccination, knowledge of the HPV genotype prevalence in the female population and its associated risk factors are important for health policy makers. Here we present data about the prevalence of HPV and HPV genotypes and associated risk factors in a randomly selected age stratified female population aged 25–65 years from Curaçao.

## 2. Material and methods

### 2.1 Study population

From the national Population Register of Curaçao, Fundashon Prevenshon, the prevention centre in Curaçao, we obtained a database containing age and ID-number of all women aged 25–65 years and registered as inhabitant of the island. From each of the age strata 25–34, 35–44, 45–54 and 55–65, 1,250 women were randomly selected. Each selected woman received an invitation letter per mail to participate in the study. If a woman did not respond, a second invitation letter was sent after 2 weeks.

In this study, we excluded all women who had a history of hysterectomy, were pregnant or less than 3 months postpartum, or had cervical (pre)cancer in the last 2 years or on-going treatment with chemo- or radiation therapy.

### 2.2 Ethical considerations and safety of participants

The Institutional Review Board of the medical ethics committee of Fundashon Prevenshon Curaçao approved the study registered with the code FP0003/15. The participants received, in systematic order and prior to their participation, detailed information about the study, the objectives and their right to interrupt the study. Documents with extensive information about the study, HPV and cancer were made available in four languages: Papiamentu (native language spoken on Curaçao), Dutch, English and Spanish. All participants signed an informed consent if they agreed to participate in the study, and before they proceed with the sample collection.

### 2.3 Questionnaire

On arrival at the prevention centre, the participants received a questionnaire in which they were asked about their age, ethnicity, and habits such as smoking, drugs- and alcohol use. Other questions of the questionnaire concerned allergies, co-morbidities, gynaecological and obstetrical background, anti-conceptive methods, sexual habits, lifetime—and current sexual partners, and sexual transmitted diseases (STDs). (S1 and S2 Appendices) This questionnaire was completed under the supervision of a nurse who, if necessary, provide additional information in case of uncertainties about the questions and also checked that the informed consent form was signed properly.

### 2.4 Specimen collection and handling

After completing the questionnaire and signing the informed consent form, the woman was referred to the research room, where the doctor took two cervical samples. First a conventional Pap-smear was collected, which was fixed with cyto-fix solution (Schubert Medizinprodukte. Wackersdorf, Germany) and stored at room temperature.

Secondly, a sample for HPV detection was collected, with the same type of brush used for cyto-collection. The sample for HPV detection was put in PreservCyt® at room temperature and at the end of each day transported to the Analytical Diagnostic Centre (ADC) Laboratory of Curaçao where the HPV samples were stored at -20°C.

### 2.5 Cytology, referred participants and histology

Smears were red by 2 cytotechnicians and scored according to the CISOE-A classification as used in The Netherlands and Curaçao. This classification can be easily translated into the Bethesda Classification.[6] In case of discrepancy, a supervising cytopathologist made the final diagnosis. All women with borderline smears or worse (comparable with ASC-US or higher), were referred to the gynaecologist for colposcopy and biopsy. The gynaecologist classified the lesion as no lesion (NEG), LSIL, HSIL or carcinoma. In all referred participants, a biopsy was taken from the lesion. In case no lesion was found during colposcopy, a blind biopsy at 12 h was taken.

Biopsy specimens for histological evaluation were read by one cytotechnician and one pathologist and classified as no lesion, CIN1, CIN2, CIN3 and carcinoma according to international criteria. [7]

### 2.6 HPV detection and genotyping

The MagnaPure 96 instrument was used for DNA isolation. Ten μL of extracted DNA was used as input for the broad spectrum GP5+/6+-PCR in a total volume of 50μL. Detection of hrHPV was done on 5μL GP5+/6+ amplimer with the Enzyme immune assay (EIA kit HPV GP HR; *Labo Bio-medical Products*, *Rijswijk*, *The Netherlands)* according to the manufacturer’s instructions. This kit detects amplified DNA from HPV genotypes 16, 18, 31, 33, 35, 39, 45, 51, 52, 56, 58, 59, 66, and 68. A 10μL aliquot of GP5+/6+ amplimer from HPV positive samples by EIA was tested with the Genotyping kit HPV GP, version 2 *(Labo Bio-medical Products)*. This kit enables genotyping of 12 hrHPV genotypes (16/ 18/ 31/ 33/ 35/ 39/ 45/ 51/ 52/ 56/ 58/ 59), 6 possible hrHPV types (26/ 53/ 73/ 82/ 66/ 68) and 5 lrHPV types (6/ 11/ 30/ 67/ 70).[8]

### 2.7 Statistical analysis

Questionnaire results are presented by frequency and percentage for categorical data and by means and standard deviations (SDs) for normally distributed continuous data. HPV type prevalence was assessed for all women and stratified by age. The association between HPV prevalence and sexual risk factors obtained via the questionnaire are tested with the chi-square test or Fisher’s exact test. In case of significant differences in risk factors between the age groups, logistic regression models are used to correct the associations between HPV prevalence and sexual risk-factors for age. A forward selection procedure (p-value to enter: p<0.05) was used to build a multivariable logistic regression model to identify all independent risk-factors associated with HPV prevalence. In a subgroup analysis, we excluded all participants who had Pap results ≥BMD (or ≥ASC-US). The significance level α was set at 0.05. SPSS version 22 (IBM Corp., Armonk, NY) was used for the statistical analyses.

## 3. Results

### 3.1 Response to the invitation

Of the 5000 invited women, 1695 women responded to participate (33.9%). Among the invited women, 310 responded but cancelled the appointment and 119 women responded but withdrew due to no interest (n = 38) or unknown reason (n = 81). Another 191 women were excluded because of hysterectomy (n = 165) and pregnancy (n = 26), leaving 1075 women for the analysis. The majority of our participants were Afro-descendants (87.2%). The sociodemographic and clinical characteristics of this population are described in Table 1.

**Table 1:** Sociodemographic characteristics and sexual behaviour.

		n	%
**Ethnicity**			
	African	937	87.2%
	Caucasian	41	3.8%
	Asian	8	0.7%
	Other	89	8.3%
**Age**			
	25–34	247	23.0%
	35–44	285	26.5%
	45–54	238	22.1%
	55–65	305	28.4%
**Sexual behaviour**			
	active	868	80.7%
	not active	202	18.8%
	no response	5	0.5%
**Number of sexual partners (current)**			
	0	258	24.0%
	1	789	73.4%
	>1	17	1.6%
	no response	11	1.0%
**Number of sexual partners (lifetime)**			
	0	4	0.4%
	1	314	29.2%
	2–5	576	53.6%
	6–10	136	12.7%
	>10	39	3.6%
	no response	6	0.6%
**Age first sexual contact**			
	≤15	110	10.2%
	16–19	598	55.6%
	≥20	361	33.6%
	not applicable*	4	0.4%
	no response	2	0.2%
	mean (SD)	19.0 (3.6)	
**Oral sex**			
	no	397	36.9%
	yes	673	62.6%
	no response	5	0.5%
**Anal sex**			
	no	758	70.5%
	yes	142	13.2%
	no response	175	16.3%
**History/current use of OAC**			
	no	298	27.7%
	yes	767	71.3%
	no response	10	0.9%
**History/current use of IUD**			
	no	846	78.7%
	yes	219	20.4%
	no response	10	0.9%
**History of STD**			
	no	951	88.5%
	yes	113	10.5%
	no response	11	1.0%
**(History of) smoking**			
	no	948	88.2%
	yes	119	11.1%
	no response	8	0.7%
**Drugs**			
	no	1057	98.3%
	yes	8	0.7%
	no response	10	0.9%

* These participants said to be virgin

### 3.2 Results of the questionnaire

The majority of the participants were sexually active (80.7%). Mean age at first sexual contact was 19.0 (SD 3.6) year. Most participants responded currently to have one sex-partner (73.4%) while a small proportion (1.6%) responded to have more than one sex-partner, and 24.0% had no sex-partner in the past twelve months. For the lifetime number of sexual partners, the majority (53.6%) had between 2 to 5 partners. Four (0.37%) participants answered they never had sex before. Most of the participants practice oral sex (62.6%) while only few (n = 142,13.2%) practice anal sex. Seven hundred and sixty seven participants (71.3%) used (or have used) oral contraceptive and 219 (20.4%) participants currently have or have been using an IUD. One hundred and thirteen (10.3%) participants mentioned history of STD.

One hundred and nineteen (11.1%) participants smoked or had smoked cigarettes. Information about the specific period exposed to cigarettes was not available. Of all participants 0.67% used any kind of recreational drugs. (Table 1)

### 3.3 Cytology and histology results in the overall population

Twenty seven participants (2.6%) were detected with abnormal cytology ≥BMD(or ≥ASC-US). (S1 Table) Eighteen of the 27 referred participants (66.7%) showed abnormal histology: one woman had an adenocarcinoma, ten women had CIN3, four had CIN2, and three had CIN1. Five referred participants had normal histology results and four did not show up for biopsy (S1 Table)

### 3.4 HPV prevalence in the overall population

HPV prevalence in the total study population was 19.7%. Multiple infections with different HPV types were detected in 42 (19.8%) women, yielding 271 infections in total, from which 70.5% were hrHPV-types and HPV 16 (2.3%) was the most common hrHPV genotype. (Table 2).

**Table 2:** HPV genotype distribution in HPV positive women in the overall population stratified by age.

Type	Age 25–34 (N = 247)		Age 35–44 (N = 285)		Age 45–54 (N = 238)		Age 55–65 (N = 305)	
	n	%	n	%	n	%	n	%
**6**	2	0.8			1	0.4	2	0.7
**11**	1	0.4						
**16**	12	4.9	5	1.8	4	1.7	4	1.3
**18**	8	3.2	5	1.8	3	1.3	3	1.0
**30**	2	0.8	1	0.4				
**31**	6	2.4	3	1.1	4	1.7	1	0.3
**33**	7	2.8	2	0.7	1	0.4	1	0.3
**35**	10	4.0	4	1.4	2	0.8	7	2.3
**39**	3	1.2	2	0.7	1	0.4		
**45**	11	4.5	3	1.1	2	0.8	2	0.7
**51**	4	1.6	1	0.4	1	0.4	2	0.7
**52**	11	4.5	1	0.4	1	0.4	6	2.0
**53**	2	0.8	1	0.4				
**56**	3	1.2	1	0.4			2	0.7
**58**	8	3.2	1	0.4	2	0.8	3	1.0
**59**	1	0.4	1	0.4	1	0.4		
**66**	2	0.8	6	2.1	5	2.1	3	1.0
**67**	2	0.8					4	1.3
**68**			1	0.4				
**70**	2	0.8	2	0.7	1	0.4		
**73**	1	0.4	1	0.4				
**82**	1	0.4	2	0.7				
**X**	14	5.7	14	4.9	11	4.6	21	6.9
**Total**	**81**	**32.8%**	**44**	**15.4%**	**36**	**14.7%**	**52**	**17.0%**
***negative***	***166***	***67*.*2%***	***241***	***84*.*6%***	***203***	***85*.*3%***	***253***	***83*.*0%***

31 participants presented 2 HPV infections, 9 presented 3- and 2 presented 6 infections. The percentage is calculated based on 212 HPV positive participants

**Fig 1:**
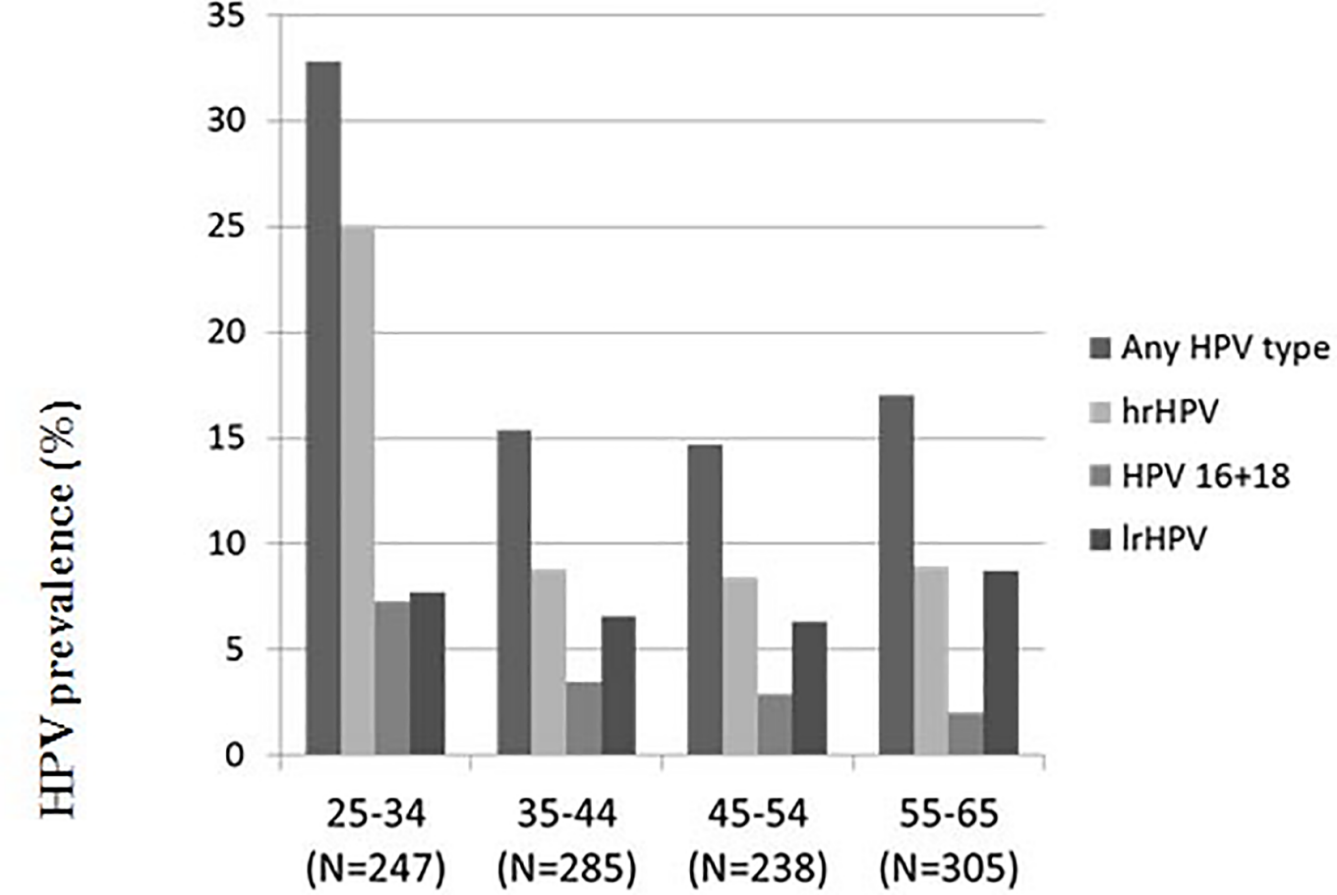
HPV genotype distribution in HPV positive women in the overall population stratified by age (both single as multiple infections included) (N = 1075). High risk HPV illustrate12 hrHPV types 16/ 18/31/ 33/ 35/ 39/ 45/ 51/ 52/ 56/ 58/ 59 and 6 possible hrHPV types 26/ 53/ 73/ 82/ 66/ 68. The vaccine types hrHPV 16,18, 31, 33, 45, 52,58 are included. Underlying numbers are presented in S2 table.

HPV prevalence differed between the age groups (p<0.001): the youngest age group had the highest prevalence (32.8%). The HPV prevalence of women in the other age groups were comparable (15.4%, 14.7% and 17.0%, in the age-groups 35–44, 45–54 and 55–65 respectively). (Fig 1)

HPV 16 (4.9%) was most common high risk genotype found in women aged 25–34 years, followed by HPV 45 (4.5%) and 52 (4.5%). In women aged 35–44 years and 45–54 years, HPV 66 (2.1% both) was the most common high risk genotype followed by HPV 16 (1.8%) and 18 (1.8%) in the age group of 35–44 years and HPV 16 (1.7%) and 31 (1.7%) in the age group of 45–54 years. In women in the age of 55–65 years, we found more of HPV 35 (2.3%), followed by HPV 52 (2.0%) and 16 (1.3%) (Table 2).

No significant difference between the age groups is observed with regard to the proportional attribution of HPV 16 (14.8% in women age 25–34 years; 11.4% in women age 35–44 years; 11.1% in women age 45–54 years and 7.7% in women age 55–64 years p = 0.22 linear by linear association) (Table 2).

In the group with abnormal cytology, 3 were HPV negative while the 3 most common genotypes in referred participants with abnormal cytology were HPV 16 (n = 9), 45 (n = 5) and 58 (n = 4).(S1 Table)

HPV prevalence of women born on Curaçao among participants (N = 826) was 20.6% and did not differ significantly from HPV prevalence of women migrated from other Caribbean islands (N = 116, 20.7% HPV-positive) and from Latin America (N = 67, 13.4% HPV-positive) (*p = 0*.*37*). (Table 3)

**Table 3:** Association between risk factors and HPV infection N = 1075.

			HPV negative		HPV positive		p-value		Age adjusted
			n	%	n	%			p-value
Country of birth***							0.37		
Curaçao			657		169	20.6%			
Caribbean islands			92		24	20.7%			
Latin America			58		9	13.4%			
Age***							**<0.001**	^$^	-
	25–34		166	67.2%	81	32.8%	** **		
	35–44		241	84.6%	44	15.4%	** **		
	45–54		203	85.3%	35	14.7%	** **		
	55–65		253	83.0%	52	17.0%	** **		
Sexual behavior***							0.060		0.17
	active		689	79.4%	179	20.6%	** **		
	not active		172	85.1%	30	14.9%	** **		
Number of sexual partners (current)***							0.14	^$^	0.41
	0		218	84.5%	40	15.5%	** **		
	1		625	79.2%	164	20.8%	** **		
	>1		15	88.2%	2	11.8%	** **		
Number of sexual partners (lifetime)***							0.30	^$^	0.50
	0		2	50.0%	2	50.0%	** **		
	1		262	83.4%	52	16.6%	** **		
	2–5		460	79.9%	116	20.1%	** **		
	6–10		103	75.7%	33	24.3%	** **		
	>10		33	84.6%	6	15.4%	** **	^ ^	
**Age first sexual contact**									
	mean (SD)		19.1 (3.7)		18.5 (3.4)		**0.030**	**	0.19
Oral sex***							0.070		0.36
	no		331	83.4%	66	16.6%	** **		
	yes		530	78.8%	143	21.2%	** **		
Anal sex***							0.58		0.75
	no		613	80.9%	145	19.1%	** **		
	yes		112	78.9%	30	21.1%	** **		
History/current use of OAC***							0.28		-
	no		233	78.2%	65	21.8%	** **		
	yes		622	81.1%	145	18.9%	** **		
History/current use of IUD***							0.59		0.21
	no		682	80.6%	164	19.4%	** **		
	yes		173	79.0%	46	21.0%	** **		
History of STD***							0.15		0.39
	no		769	80.9%	182	19.1%	** **		
	yes		85	75.2%	28	24.8%	** **		
(History/current) smoking***							0.62		-
	no		767	80.9%	181	19.1%	** **		
	yes		94	79.0%	25	21.0%	** **		
Drugs***							0.19	*	-
	no		854	80.8%	203	19.2%	** **		
	yes		5	62.5%	3	37.5%	** **		

^$^ Linear-by-Linear association

* Via Fisher’s exact test

** Via independent samples t-test

- not corrected for age; no significant difference between age groups.

***Total do not sum 1075 in case of unanswered questions.

A subgroup analysis of women with normal cytology (n = 1.048) showed 188 HPV positive participants (17.9%). In contrast with the overall population the attribution of the different HPV genotypes changed in ranking order to: HPV 35 (2.0%), 18(1.8%), 16 and 52, each (1.5%) as the most common types. (S2 Table)

### 3.5 Association between HPV prevalence and sexual risk factors and gynaecological history

HPV prevalence was found to be significantly associated with young age and age at first sexual intercourse. The mean age of first sexual contact of HPV-positive women was 18.5 (SD 3.4) year and of HPV-negative women was 19.1 (SD 3.7) year (p = 0.032). However, after adjusting for age, this risk factors was not significantly associated with HPV prevalence. (Table 3) In the forward selection procedure, only age was entered.

## Discussion

**Fig 2:**
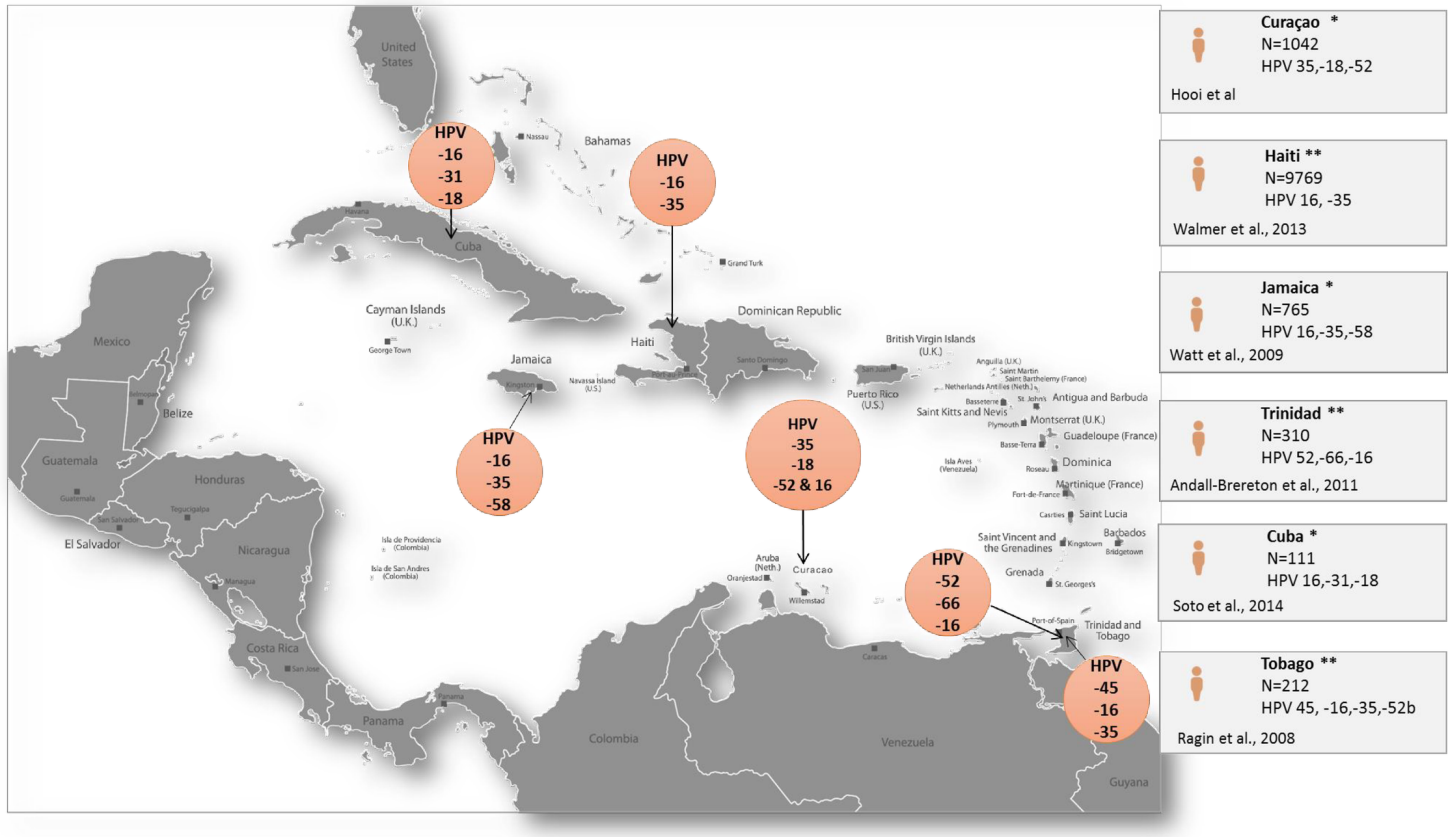
Illustration of the HPV type distribution in women with normal cytology. Each study used different methods to:—gather the studied population, -determine HPV in lab, statistically analyse the results and to describe the data. Distribution of the most common HPV genotypes in Guadeloupe are not described in this figure as in the publication these are not specified. *HPV prevalence in population with normal cytology. **HPV prevalence in population with normal and abnormal cytology.

HPV prevalence in the total study population of Curaçao was high (19.7%) and is comparable with the prevalence on some other islands in the Caribbean, i.e. Haiti (19.0%) and Guadeloupe (25.1%) [9,10] (Fig 2) However, two studies from Jamaica (HPV prevalence 87.7% and 50.9% respectively) and another from Trinidad (HPV prevalence 40.6%), showed an even higher HPV prevalence. [11,12,13] When women with abnormal cytology were excluded from our analysis, an HPV prevalence of 17.8% was found. Compared to the standard HPV prevalence in the world population (4.1%) this HPV prevalence is high, but in line with overall HPV prevalence data from Caribbean Islands of 15.8% as given by ICO’s world report. [4]

The most frequent HPV types in the total population of Curaçao were HPV 16 (2.3%), 35 (2.1%), 18 (1.8%) and 52 (1.8%). This type distribution is largely comparable with a study from Jamaica (ranking HPV 16, 35, 58) and one from Haiti (HPV 16 and 35) [9,12]. However for women from Trinidad, the most common genotypes were reported to be HPV 52, 66,16. [13]

HPV genotype prevalence data are difficult to compare with other Caribbean data because of differences in HPV genotyping assays used. Moreover, interpretation is hampered because women in these studies were derived from different populations i.e. screening populations, gynaecologic outpatient populations or a mixture of these, and consequently include varying proportions of women with abnormal cytology. This has a large influence on HPV positivity and genotype distribution in these populations, because of different preferential risks for CIN 2/3 lesions and cancer of different HPV types. To encompass this problem, we also studied HPV prevalence and HPV genotype distribution in women with normal cytology. In women with normal cytology from Curaçao the most commonly detected HPV genotypes were: HPV 35 (1.9%), 18 (1.8%), 16 and 52 (each 1.5%). [14] Only from Guadeloupe a study in women with normal cytology was published (n = 447). The authors found an hrHPV prevalence of 25.1%. Although the non16 and18 HPV genotypes were not specified in this study they also found a higher prevalence of non16 and18 HPV types in the studied population (HPV 16 and 18: 5.4% (24/447) versus non16 and18 HPV genotypes 19.7% (88/447 p = 0.004). [10]

Interestingly in Curaçao, the HPV genotype distribution in the population with normal cytology differs from the HPV genotype distribution in women with cervical cancer: HPV 16 shifts in ranking from 3^rd^ to 1^st^ place, whereas HPV 35 which was the most prevalent in normal cytology was rare in cervical cancer specimens. HPV45, which is not commonly seen in women with normal cytology from Curaçao appears in the three most common HPV types associated with cancer. HPV 45 is often associated with adenocarcinoma of the cervix which is localised higher in the endocervical canal and this may explain why infection with HPV 45 is not easily detected in exfoliated cells of women with normal cytology and cervical precursor lesions. [15] An alternative explanation may be that in women from Curaçao HPV 45 has a higher preferential risk for adenocarcinoma, a phenomenon earlier described by Bulk et al. in a study conducted in the Netherlands in 2005.[16]

In this survey, only young age, and early age at sexual debut were found to be statistically associated with a high HPV prevalence as has been described by others.[17,18]

In our questionnaire only 10.5% of women reported having a history of any type of STD. Probably, a higher number of participants had been infected with an STD in the past, but were unaware that they had contracted an STD because they were not familiar with the recognition of symptoms associated STD’s. Also, 4 participants referred they never had sexual contact while 2 of them were HPV positive. (Table 3)

A strong point of our study is that to the best of our knowledge, this is the first attempt in the Caribbean area to describe HPV prevalence and HPV-genotype distribution in relation to sexual behaviour and other risk factors in a randomly selected population of women of all age groups (25–65 years). Moreover, the HPV prevalence and HPV genotype distribution were studied in both women from the total population and women with normal cytology. Another strong point is that one person at one centre collected all samples. In the laboratory, one technician was responsible for the different handling procedures, and evaluated all Pap-smears whereas another technician performed all HPV assays, unaware of the results of the cytology report. All the work was realized over a relatively short period of 3–4 months. A limitation of our study is that our studied population was based on participants 25 years and above while HPV prevalence in the population <25 years is probably higher than in the studied population.

In conclusion, we showed in a randomly selected population of women (age 25–65) from Curaçao that HPV prevalence is high, and very high in women of 25–34 years. These HPV prevalence data ask for the introduction of prevention strategies. HPV 16 is the most common HPV type detected in the overall population eligible for cervical screening whereas HPV 35 is the most commonly detected HPV type in women with normal cytology. Young age and early age of sexual debut were the only risk factors associated with a high HPV prevalence.

Together with our earlier published HPV genotyping findings in cervical cancer these HPV genotype data in the population of Curaçao should be taken into account in the choice of a prophylactic HPV vaccine when a vaccination programme is implemented in Curaçao.

## Supporting information

S1 Appendix Questionnaire form in Papiamentu. (DOCX)

S2 Appendix Questionnaire form in English. (DOCX)

S1 Table Women referred for abnormal cytology ≥BMD (ASC-US) and colposcopy and histology results in follow-up. * Data not available BD borderline dyskariosis, MD Mild dyskariosis are comparable to ASCUS ASCUS–H/LSIL.
(DOCX)

S2 Table HPV type attribution in HPV positive women with normal cytology and total studies population. 28 participants presented 2 HPV infections, 6 presented 3- and 2 presented 6 infections. * The percentage is calculated based on all participating women (n = 1075). ** The percentage is calculated based on 1.048 women excluding all participants with ≥ BMD (ASC-US) or more.
(DOCX)
