# Supplementary material for: Human papillomavirus (HPV) prevalence and associated risk factors in women from Curaçao
Source: PLoS One. 2018 Jul 13;13(7):e0199624. doi: 10.1371/journal.pone.0199624 (PMC6044524; doi:10.1371/journal.pone.0199624)
Supplement: S1 Appendix — (DOCX) [file pone.0199624.s001.docx]

**
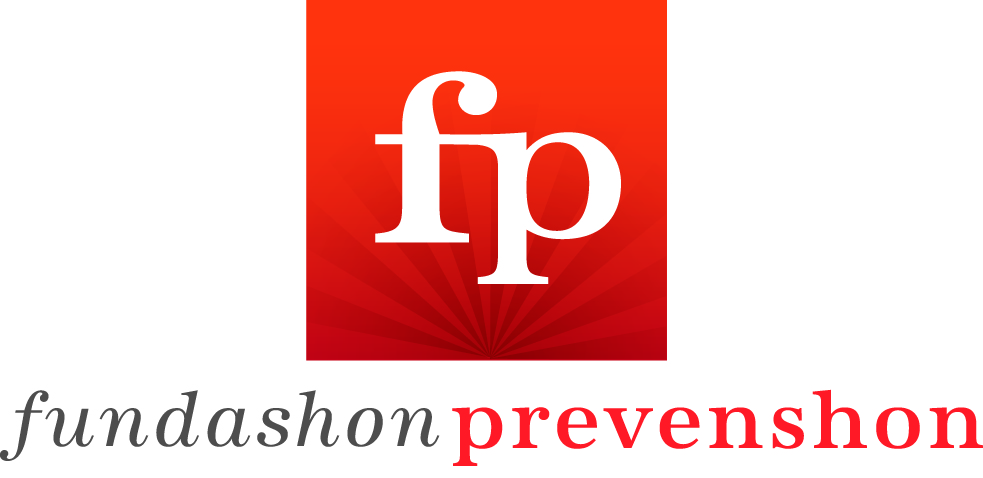
**

**Formulario di Anamnesis**

**Datos Personal**

Number di investigashon:………………………

Number di sedula:………………………………….

- Nòmber:………………………………………………….
- Fam:………………………………………………………..
- Edat:………………………………………………………..
- Etnia:……………………………………………………….
- Fecha di nasementu:……………………………….
- Lugá di nasementu:…………………………………
- Direkshon:……………………………………………….
- Bario:……………………………………………………….
- Number di telefòn:………………………………….
- Bibá na Korsou for di aña:……………………..
- Dòkter di kas:………………………………………….

Marka un krus pa e kontesta korekto

- **Kustumber**
- Huma SI / NO
- Alkohòl SI / NO
- Droga SI / NO
- Si ta usa droga kua tipo
- **Alergía**
- Jodo SI / NO
- Hanskun(latex) SI / NO
- Pa otro kos SI / NO
- **Enfermedat kròniko**
- **1**
- **2**
- **3**
- **Uso di medikamento**
- Nòmber mg dd
- Nòmber mg dd
- Nòmber mg dd
- Nòmber mg dd
- **Historia ginekológiko**
- Edat di promé menstruashon/regla……………………………….
- Edat di promé relashon seksual……………………………………..
- Fecha di último relashon seksual…………………………………….
- Frekuensha di relashon seksual……………………………………….
- Sekso oral? Si / NO
- Sangramentu durante kontakto seksual? Si / NO
- Sangramentu mei mei di 2 periodo di menstruashon SI / NO
- Sangramentu despues di menopousa SI / NO

Kantidat di pareha seksual te awor……………………

Kantidat di pareha seksual actual………………………

- Kantidat di embaraso………………………………………….
- Kantidat di joe
- Abortus SI/NO
- Ki tipo di protekshon ta usa kontra di embaraso òf kontra enfermedadnan di transmishon seksual?......................................
- Kon largu a usa esaki i for di ki tempu a stop di usa?.....................
- Fecha di último menstruashon/regla……………………………………………………
- Kon e siklo di menstruashon/regla ta of tabata? Regular / Iregular
- Fecha di último Pap?...................................
- Resultado?.......................
- Kiko dòkter a palabra despues di e resultado aki……………………………….
- Tabatin enfermedad di transmishon seksual den pasado? SI / NO
- Si ta asina, spesifiká kua, na ki aña, ki tratamentu a risibí pa esaki, i si a kontrolá despues…………………………………………………………………………
- ……………………………………………………………………………………………………………..

**Ta konosí ku tratamentu dor di un ginekólogo/ of operashon dor di un ginekólogo?** SI / NO
Si ta asina, kiko ta e motibu?......................................................

For di kua fecha……………………………………………………………………………………

Geheimhoudingsdocument

Hierbij verklaart ……………………………………………, dat ik de bovengenoemde vragen uit alle eerlijkheid heb beantwoord. Ik heb geen bezwaar om de bovengenoemde medische ingrepen te ondergaan.

Firma di dokter Firma di kliente

……………………………………. …………………………………………

Alleen voor de arts

***Consult of Verwijzing?***

*Reden van consult:*

*Reden van verwijzing:*

***Medische Handeling***

*PAP test*

*HPV zelf screening*

*HPV screening*

*Orale HPV screening*

*Colposcopie*

*Biopsie*

*LLEDZ*

***Observaties***

***Beleid***

***Afspraken***
