# Supplementary material for: Human papillomavirus (HPV) prevalence and associated risk factors in women from Curaçao
Source: PLoS One. 2018 Jul 13;13(7):e0199624. doi: 10.1371/journal.pone.0199624 (PMC6044524; doi:10.1371/journal.pone.0199624)
Supplement: S2 Appendix — (DOCX) [file pone.0199624.s002.docx]

-
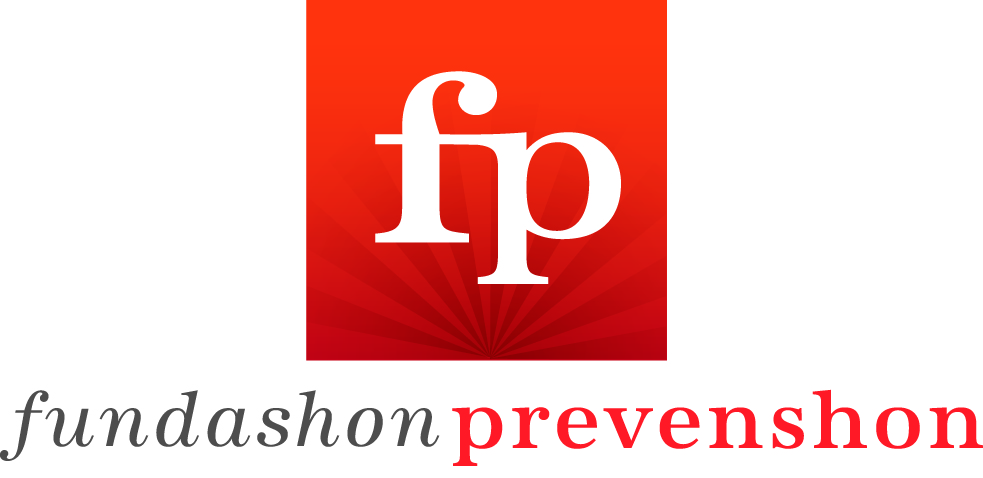


  **History form**
  Research Number: .....................................................................
  Identity: .......................................................................................
  Name: .........................................................................................
  Last Name: .................................................................................
  Age: ............................................................................................
  Ethnicity: .....................................................................................
  Date of Birth: ..............................................................................
  Place of Birth: ............................................................................
  Residential Address: ..................................................................
  Neighborhood: ............................................................................
  Phone Number: ..........................................................................
  Lives in Curaçao since: ..............................................................
  GP: .............................................................................................
  Mark the correct answer

  **Habits**Smoking: YES / NO
  Alcohol: YES / NO
  Drugs: YES / NO
  If yes, what sort?
   **Allergies**Jodium: YES / NO
  Gloves (latex): YES / NO
  Others: YES / NO
   **Chronic diseases**1………………………………………………………………..
  2………………………………………………………………..
  3………………………………………………………………..

**Medications**

- Name ………………… mg dd
- Name mg dd
- Name mg dd
- Name mg dd
- **Gynecological history**First menstruation:……………………………………..
  First sexual contact:……………………………………
  Last sexual contact: ……………………………………
  Sex frequency: ……………………………………………
  Oral sex? YES / NO
  Bleeding during sex: YES / NO
  Bleeding between the menstrual cycle: YES / NO
  Bleeding after menopause: YES / NO

  Number of partners during lifetime? ………………………………….

Number of partners; actual ………………………………………………..

- Number of pregnancies?..............................................
- Number of children?....................................................
- Abortion: YES / NO
- Preservative: YES / NO
  If your answer is yes
  Type of preservative:…………………………………………………………….
  Active since what date:………………………………………………………….
- Duration:………………………………………………………………………………..
- Last menstruation:………………………………………………………………..
- How is or was your menstrual cycle: Regular / Irregular
- Last Pap smear: ……………………………………………………………………
- Results: ………………………………………………………………………………..
- Agreements with the doctor after the last Pap smear:………
- ……………………………………………………………………………………………….

  Sexual transmitted disease? ………………………………………………..
- Gynecological issues/ gynecological surgical procedures? YES/NO

Specify issue or procedure: ……………………………………………………………………… Treatingdoctor......................................................................
Date of procedure………………………………………………………………..……………………….

Medical purposes ***Medical procedures***

*PAP smear*

*HPV self sample*

*HPV test*

*Orale HPV test*

*Colposcopy*

*Biopsy*

*LLEDZ*

***Observations***

***Policy***

***Appointments***
