# Supplementary material for: Human papillomavirus (HPV) prevalence and associated risk factors in women from Curaçao
Source: PLoS One. 2018 Jul 13;13(7):e0199624. doi: 10.1371/journal.pone.0199624 (PMC6044524; doi:10.1371/journal.pone.0199624)
Supplement: S1 Table — * Data not available BD borderline dyskariosis, MD Mild dyskariosis are comparable to ASCUS ASCUS–H/LSIL. (DOCX) [file pone.0199624.s003.docx]

**S 1.**

|  | Cytology | HPV | Colposcopy | CIN |
| --- | --- | --- | --- | --- |
| 1 | 2 (BMD) | 45 | NEG | NEG |
| 2 | 2 | 35, 67 | NEG | NEG |
| 3 | 2 | 66 | NEG | * |
| 4 | 2 | x | LSIL | NEG |
| 5 | 2 | 45 | LSIL | CIN 1 |
| 6 | 2 | NEG | LSIL | CIN 2 |
| 7 | 2 | 31, 58, 66 | LSIL | CIN 2 |
| 8 | 3a (BMD) | 16 | LSIL | NEG |
| 9 | 3a | 51 | LSIL | NEG |
| 10 | 3a | 33 | LSIL | CIN 1 |
| 11 | 3a | 51 | LSIL-HSIL | CIN 1 |
| 12 | 3a | 31, 52 | NEG | CIN 2 |
| 13 | 3a | 58 | LSIL | CIN 2 |
| 14 | 3a | 16 | HSIL | CIN 3 |
| 15 | 3a | 16 | * | * |
| 16 | 3a | NEG | * | * |
| 17 | 3b (SD) | 16 | LSIL | CIN 3 |
| 18 | 3b | NEG | HSIL | CIN3 |
| 19 | 3b | 58 | HSIL | CIN 3 |
| 20 | 3b | 16, 45, 52 | HSIL | CIN 3 |
| 21 | 3b | 16, 45, 52 | HSIL | CIN 3 |
| 22 | 3b | 35 | HSIL | CIN 3 |
| 23 | 3b | 16 | * | * |
| 24 | CIS | 58, 66 | LSIL-HSIL | CIN 3 |
| 25 | 4 | 16 | HSIL | CIN 3 |
| 26 | Carcinoma | 16 | HSIL | CIN 3 |
| 27 | 5 | 45 | Invasive |  |
