# Supplementary material for: Human papillomavirus (HPV) prevalence and associated risk factors in women from Curaçao
Source: PLoS One. 2018 Jul 13;13(7):e0199624. doi: 10.1371/journal.pone.0199624 (PMC6044524; doi:10.1371/journal.pone.0199624)
Supplement: S2 Table — 28 participants presented 2 HPV infections, 6 presented 3- and 2 presented 6 infections. * The percentage is calculated based on all participating women (n = 1075). ** The percentage is calculated based on 1.048 women excluding all participants with ≥ BMD (ASC-US) or more. (DOCX) [file pone.0199624.s004.docx]

**S 2.**

|  | **Total studied population N=1075** | | **Only normal cytology N=1048** | |
| --- | --- | --- | --- | --- |
| type |  |  |  |  |
|  | n | %* | n | %** |
| 6 | 5 | 0.5 | 5 | 0.5 |
| 11 | 1 | 0.1 | 1 | 0.1 |
| 16 | 25 | 2.3 | 16 | 1.5 |
| 18 | 19 | 1.8 | 19 | 1.8 |
| 30 | 3 | 0.3 | 3 | 0.3 |
| 31 | 14 | 1.3 | 12 | 1.1 |
| 33 | 11 | 1.0 | 10 | 1.0 |
| 35 | 23 | 2.1 | 21 | 2.0 |
| 39 | 6 | 0.6 | 6 | 0.6 |
| 45 | 18 | 1.7 | 13 | 1.2 |
| 51 | 8 | 0.7 | 6 | 0.6 |
| 52 | 19 | 1.8 | 16 | 1.5 |
| 53 | 3 | 0.3 | 3 | 0.3 |
| 56 | 6 | 0.6 | 6 | 0.6 |
| 58 | 14 | 1.3 | 10 | 1.0 |
| 59 | 3 | 0.3 | 3 | 0.3 |
| 66 | 16 | 1.5 | 13 | 1.2 |
| 67 | 6 | 0.6 | 5 | 0.5 |
| 68 | 1 | 0.1 | 1 | 0.1 |
| 70 | 5 | 0.5 | 5 | 0.5 |
| 73 | 2 | 0.2 | 2 | 0.2 |
| 82 | 3 | 0.3 | 3 | 0.3 |
| X | 60 | 5.6 | 59 | 5.6 |
| **Total pos.** | **212** | **19.7** | **188** | **17.9** |
| ***Total neg.*** | ***863*** | ***80.3*** | ***860*** | ***82.1*** |
